# Mutations in *TAC1B* drive increased *CDR1* and *MDR1* expression and azole resistance in *Candida auris*

**DOI:** 10.1128/aac.00300-25

**Published:** 2025-08-18

**Authors:** Katherine S. Barker, Darian J. Santana, Qing Zhang, Tracy L. Peters, Jeffrey M. Rybak, Joachim Morschhäuser, Christina A. Cuomo, P. David Rogers

**Affiliations:** 1 Department of Pharmacy and Pharmaceutical Sciences, St. Jude Children's Research Hospital 5417, Memphis, Tennessee, USA; 2 Institute of Molecular Infection Biology, University of Würzburg 9190, Würzburg, Germany; 3 Department of Molecular Microbiology and Immunology, Brown University 6752, Providence, Rhode Island, USA; 4 Broad Institute 33577, Cambridge, Massachusetts, USA; University of Iowa 4083, Iowa City, Iowa, USA

**Keywords:** *TAC1B*, *Candida auris*, fluconazole, resistance, *CDR1*

## Abstract

*Candida auris* has emerged as a fungal pathogen of particular concern owing in part to its propensity to exhibit antifungal resistance, especially to the commonly prescribed antifungal fluconazole. A mutation in *TAC1B*, which encodes a zinc cluster transcription factor, has been shown to confer increased resistance to fluconazole. In this work, we aimed to determine how mutations in *TAC1B* exert this effect. *TAC1B* mutations leading to A640V, A657V, and F862_N866del, found in fluconazole-resistant clinical isolates, were introduced into two susceptible Clade I backgrounds using CRISPR-Cas9 gene editing. These *TAC1B* mutations conferred increased fluconazole resistance, as well as increased resistance to other triazoles as measured by broth microdilution. RNA-seq revealed that the ATP-binding cassette (ABC) transporter gene *CDR1* as well as the major facilitator superfamily (MFS) transporter gene *MDR1* were both upregulated in the presence of these *TAC1B* mutations. Disruption of *CDR1* increased susceptibility in strains with *TAC1B* mutations, whereas disruption of *MDR1* had little to no effect. However, disruption of both *CDR1* and *MDR1* resulted in an additional increase in susceptibility as compared with disruption of *CDR1* alone. *TAC1B* mutations, leading to A640V, A657V, and F862_N866del all result in increased resistance to fluconazole and other triazole antifungals and increased expression of both *CDR1* and *MDR1* in *C. auris*. Together, these data suggest *CDR1* is the primary driver of resistance conferred by these *TAC1B* mutations.

## INTRODUCTION

Since its initial identification in 2009, *Candida auris* has emerged as an important healthcare-associated fungal pathogen causing outbreaks worldwide (1, 2). Of particular concern is the prevalence of antifungal resistance among *C. auris* isolates, including resistance to two and sometimes all three antifungal classes currently available for treatment of serious *Candida* infections (3, 4). Strikingly, over 90% of isolates exhibit resistance to the most widely prescribed antifungal worldwide, the triazole antifungal fluconazole.

Fluconazole exerts its antifungal activity by competitively binding to and inhibiting lanosterol 14α-demethylase, a key enzyme of the fungal sterol biosynthesis pathway. In *Candida* species, this results in both reduced production of the major membrane sterol ergosterol as well as accumulation of a toxic sterol intermediate (5). Fluconazole resistance in *Candida* species can be the result of mutations in the *ERG11* gene, which encodes lanosterol 14α-demethylase, resulting in altered drug binding or enhanced preference for the natural substrate, leading to reduced enzyme inhibition (6). Resistance can also be due to mutations in the genes encoding the transcriptional regulators Tac1, Mrr1, or Upc2, which result in overexpression of genes encoding the ATP-binding cassette (ABC) transporter Cdr1, the major facilitator superfamily (MFS) transporter Mdr1, or Erg11, respectively (7–9). Rarely, loss-of-function mutations are found in the sterol desaturase gene *ERG3* conferring resistance by abrogating the need for lanosterol 14α-demethylase (10).

Three predominant *ERG11* mutations, leading to amino acid substitutions Y132F, K143R, and VF125AL, have been shown to contribute to fluconazole resistance in *C. auris* (11). The transcription factor Mrr1 and the MFS transporter Mdr1 have also been implicated in resistance in isolates from Clade III (12). We have previously shown that *CDR1* deletion in a resistant isolate that overexpresses this gene leads to a significant reduction in resistance to triazole antifungals (13). We subsequently identified mutations in the gene encoding the transcription factor Tac1B in resistant isolates that were evolved *in vitro* in the presence of fluconazole and documented the most common *TAC1B* mutations among resistant clinical isolates, namely, those leading to the A640V and A657V amino acid substitutions, and F682_N866del (14). Further, we demonstrated that the mutation leading to the A640V substitution in and of itself confers increased fluconazole resistance (14). However, the relationship between mutations in *TAC1B*, *CDR1*, or other potential resistance effectors remains unclear as the effect of *TAC1B* mutations on the expression of *CDR1* or other genes has yet to be investigated. In the present study, we establish that the *TAC1B* mutations leading to A640V, A657V, and F862_N866del all result in increased resistance to triazole antifungals, as well as increased expression of both *CDR1* and *MDR1* in *C. auris*. We also show that *CDR1* is the primary driver of resistance conferred by these *TAC1B* mutations.

## RESULTS

### *TAC1B* mutations observed in clinical isolates confer fluconazole resistance and reduced susceptibility to other triazole antifungals

Previously, we showed that introduction of the *TAC1B* mutation leading to the A640V substitution into susceptible clinical isolate AR0387 results in an increase in fluconazole MIC from 1 to 8 µg/mL, and correction of this mutation to the wild-type sequence in resistant clinical isolate AR0390 reduces fluconazole MIC from 256 to 16 µg/mL (14). The A640V substitution is the most common mutation we observed among Clade I isolates, and the F862_N866del in-frame deletion and A657V substitution are the first and second most common among Clade IV isolates. *TAC1B* mutations in Clade II isolates are uncommon, and Clade III isolates are not known to carry *TAC1B* mutations. In order to further investigate the contribution of *TAC1B* mutations to triazole antifungal resistance, we introduced A640V, A657V, and F862_N866del (referred to as “ADdel” for “Activation Domain deletion”) into *C. auris* strain 1c. Strain 1c is derived from highly fluconazole-resistant Clade Ic clinical isolate Kw2999 (fluconazole MIC = 256 µg/mL) that harbors an *ERG11* mutation, leading to the K143R substitution and the *TAC1B* mutation, leading to the A640V substitution (15). In strain 1c, both mutations have been corrected to their wild-type sequences, resulting in fluconazole MIC of 2 µg/mL. Reintroduction of the A640V substitution in 1c (strain 1cA640V) resulted in an increase in fluconazole MIC to 32 µg/mL. Introduction of the A657V substitution (strain 1cA657V) likewise increased the MIC to 32 µg/mL, whereas F862_N866del (strain 1cADdel) increased the MIC to 64 µg/mL (Table 1). The susceptibilities to voriconazole, isavuconazole, itraconazole, and posaconazole were also reduced (Table 1). These results indicate that each of these *TAC1B* mutations confers increased resistance to the triazole antifungals.

**TABLE 1:** Fluconazole MIC values^a^ of parental strain 1c and its *TAC1B* mutant derivative strains

	Fluconazole	Itraconazole	Posaconazole	Voriconazole	Isavuconazole
1c	2	0.031	0.0625–0.125	0.015	0.0038–0.0078
1cA640V					
Clone 1	32	0.25-0.5	0.125–0.25	0.25	0.125–0.25
Clone 2	32	0.5	0.25	0.25	0.25
1cA657V					
Clone 1	32	0.25	0.125	0.25	0.125
Clone 2	32	0.5	0.125–0.25	0.25-1	0.125–0.25
1cADdel					
Clone 1	64	0.25-0.5	0.125–0.25	0.5	0.5
Clone 2	64	0.5	0.125–0.25	0.5	0.5

^a^ MIC values are in µg/mL and represent at least three biological replicates.

### Mutations in *TAC1B* drive overexpression of *CDR1* and *MDR1*

**Fig 1:**
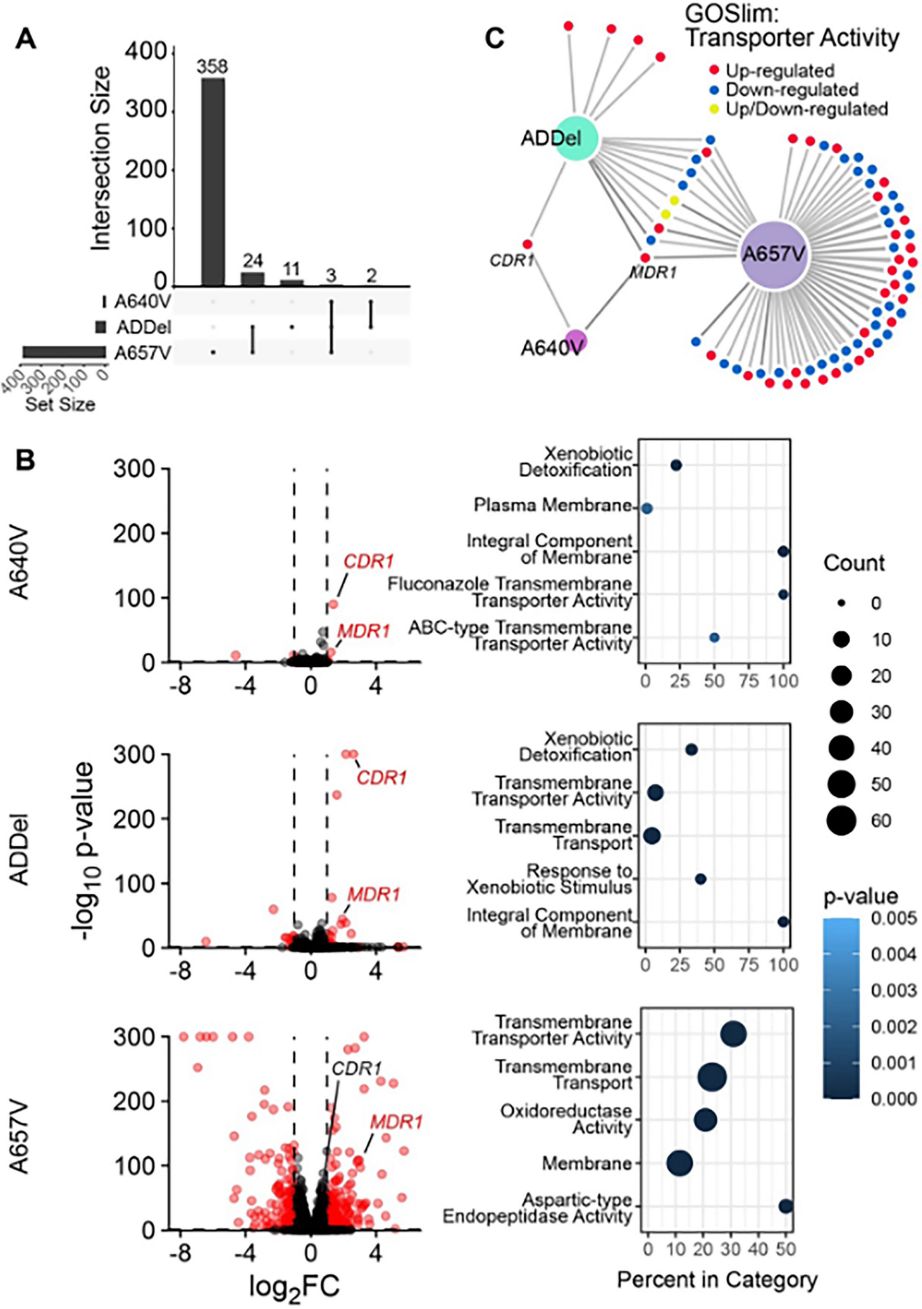
(**A**) UpSet plot of transcriptional network overlap, demonstrating the intersections of genes that are dysregulated by >2-fold and adjusted *P*-value of <0.05 compared with the parental isolate for each strain harboring different *TAC1B* mutations. Each bar in the upper region signifies the number of dysregulated genes In each intersection, the lower region signifies which strains exhibit dysregulation in those genes. (**B**) Volcano plots demonstrate magnitude of gene dysregulation for each strain, with significantly dysregulated genes colored in red. The top five overrepresented gene ontology terms in each data set are listed. (**C**) All dysregulated genes that match the GOSlim term for Transporter Activity are represented for each strain. Colors represent the directionality of dysregulation. For all panels, strains are indicated by their harbored *TAC1B* mutation, while “ADdel” refers to the F862_N866del mutation.

As there were increases in azole resistance for each mutant, we investigated whether there was a common transcriptional response. We performed RNA-seq on strain 1c and clone A from each *TAC1B* mutant strain and established a cutoff for significant gene dysregulation at twofold compared with strain 1c with a DeSeq2 adjusted *P*-value of <0.05. We observed an overlapping set of dysregulated genes among the *TAC1B* mutant backgrounds, but each also showed substantial differences in transcriptional response. For instance, the A640V mutation resulted in only five dysregulated genes, each of which was also represented by either A657V or F862_N866del (ADdel) (Fig. 1A). Of the 40 ADdel mutant dysregulated genes, 72.5% were dysregulated in at least one of the other two strains (Fig. 1A). Interestingly, the A657V mutation resulted in a large unique transcriptional response, with 358 genes being dysregulated only in this mutant (Fig. 1A). Gene Ontology Enrichment analysis revealed similar patterns of enriched gene sets between strains, with common terms surrounding transmembrane transport and xenobiotic detoxification frequently represented, in line with the role of *TAC1* homologs in other species in regulating transporter and efflux activity (Fig. 1B). Among these were efflux pumps *MDR1*, upregulated in all three mutants, and *CDR1*, upregulated in the A640V and ADdel mutants. In the A657V mutant, *CDR1* showed a 1.7-fold increase in expression. While this did not meet the fold-change cutoff for significance, this increase may still be sufficient to functionally explain the observed increase in triazole MIC (Fig. 1B). Dozens of other genes with predicted transporter function were also dysregulated in this strain background, raising the possibility of other influential effectors (Fig. 1C).

**Fig 2:**
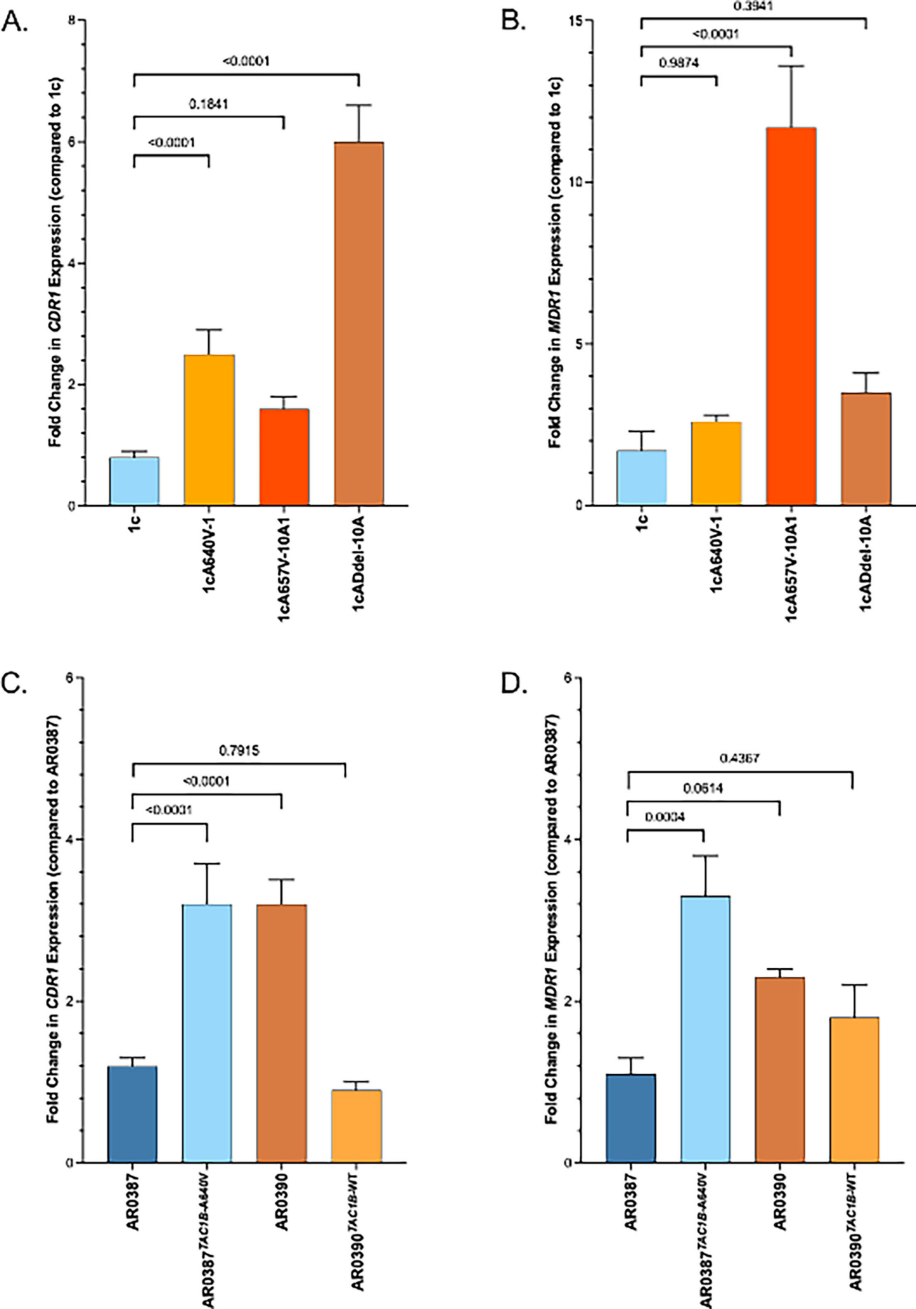
Fold change in *CDR1* (**A**) and *MDR1* (**B**) RNA expression for 1c *TAC1B* mutant strains and *CDR1* (**C**) and *MDR1* (**D**) transcript abundance for the AR0387 and AR0390 *TAC1B* allele swap strains was measured by qRT-PCR. In each graph, the median dCT value (calculated from the average *ACT1* gene CT value from three technical replicates subtracted from the average target gene CT value from three technical replicates) from three biological replicates of strain 1c or isolate AR0387, for **A and B** and **C and D**, respectively, was the comparator. Statistical significance of pair-wise comparisons is indicated by *P*-values given.

We then measured *CDR1* and *MDR1* expression by qRT-PCR in strains 1cA640V, 1c657V, and 1cADdel compared with strain 1c and observed 2.5-, 1.6-, and 6-fold increase in *CDR1* expression (Fig. 2A) and 2.6-, 11.7-, and 3.5-fold increase in *MDR1* expression, respectively (Fig. 2B). We also measured *CDR1* and *MDR1* expression by qRT-PCR in susceptible clinical isolates AR0387, its derivative carrying the A640V substitution, resistant clinical isolate AR0390, and its derivative where the A640V mutation has been corrected to the wild-type sequence. *CDR1* and *MDR1* were upregulated 3.2-fold and 3-fold, respectively, in the presence of the A640V substitution in the AR0387 background (Fig. 2C and D). Likewise, expression of these genes was reduced to wild-type levels when the A640V substitution was corrected to the wild-type sequence (Fig. 2C and D). These data indicate that *TAC1B* mutations drive *CDR1* and *MDR1* overexpression in *C. auris*-resistant isolates.

### *TAC1B***-**mediated fluconazole resistance is driven predominantly by *CDR1* overexpression with a lesser contribution from *MDR1*

**Fig 3:**
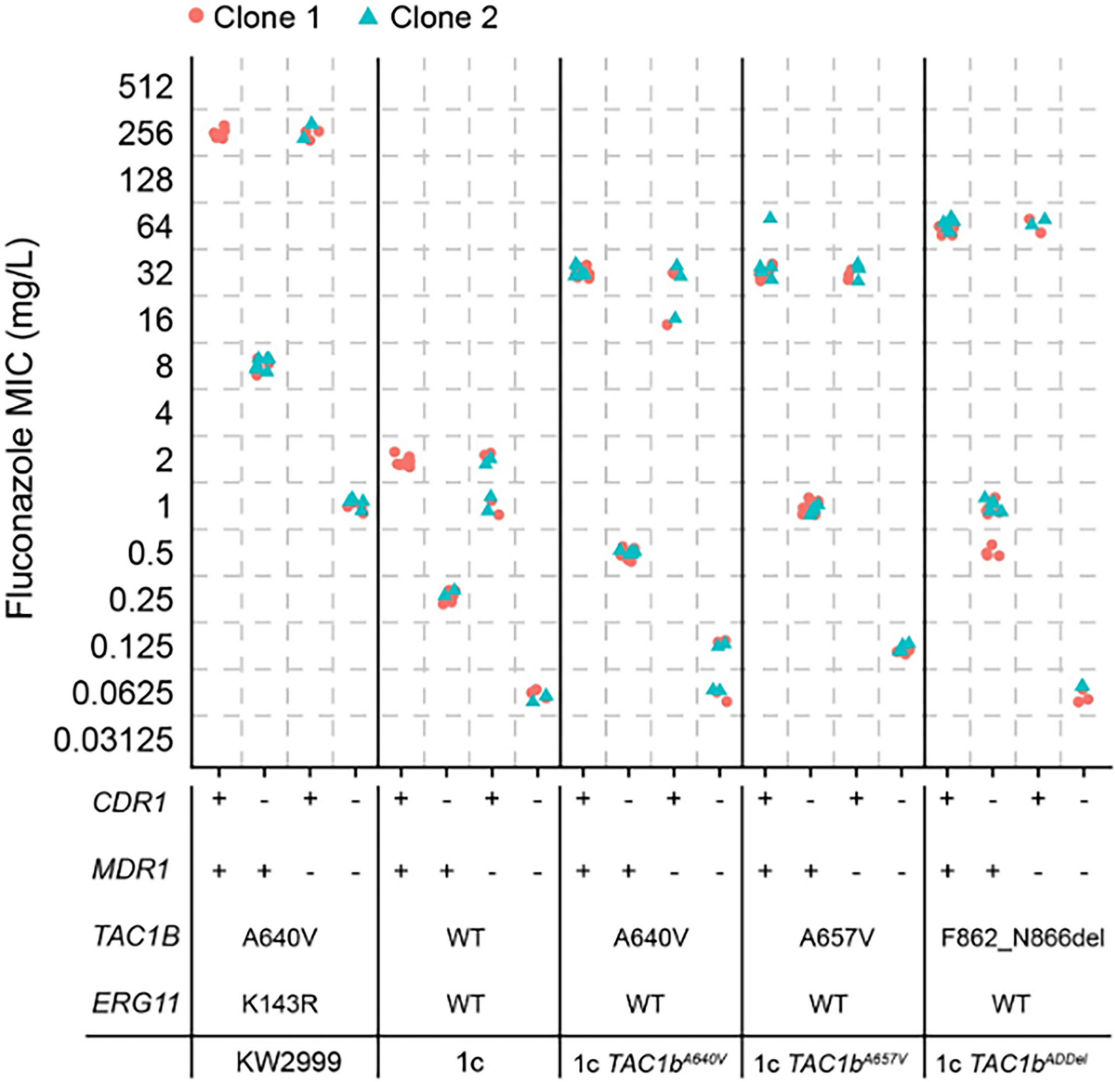
Fluconazole MICs were measured by broth microdilution in strains in which *CDR1* and *MDR1* were truncated by premature stop codon individually or in combination in Kw2999, 1c, or derivative backgrounds harboring indicated *TAC1B* mutations. Two independent clones were generated for each mutant combination. Each point represents a distinct MIC experimental replicate.

In order to further examine the contribution of *CDR1* and *MDR1* to fluconazole resistance due to *TAC1B* mutations, we disrupted these genes in resistant clinical isolate Kw2999 and strain 1c. *CDR1* disruption in Kw2999 resulted in a reduction in fluconazole MIC from 256 to 8 µg/mL, and disruption in 1c resulted in a reduction in fluconazole MIC from 2 to 0.25 µg/mL. *MDR1* disruption in isolate Kw2999 or strain 1c, however, had no effect on fluconazole MIC (Fig. 3). Disruption of *CDR1* in strain 1cA640V resulted in a decrease in fluconazole MIC from 32 to 0.5 µg/mL, disruption in 1cA657V reduced the MIC from 32 to 1 µg/mL, and disruption in 1cADdel reduced the MIC from 64 to 0.5–1 µg/mL. As in Kw2999 and 1c, *MDR1* disruption had no effect on fluconazole MIC in any of these three strains (Fig. 3).

**Fig 4:**
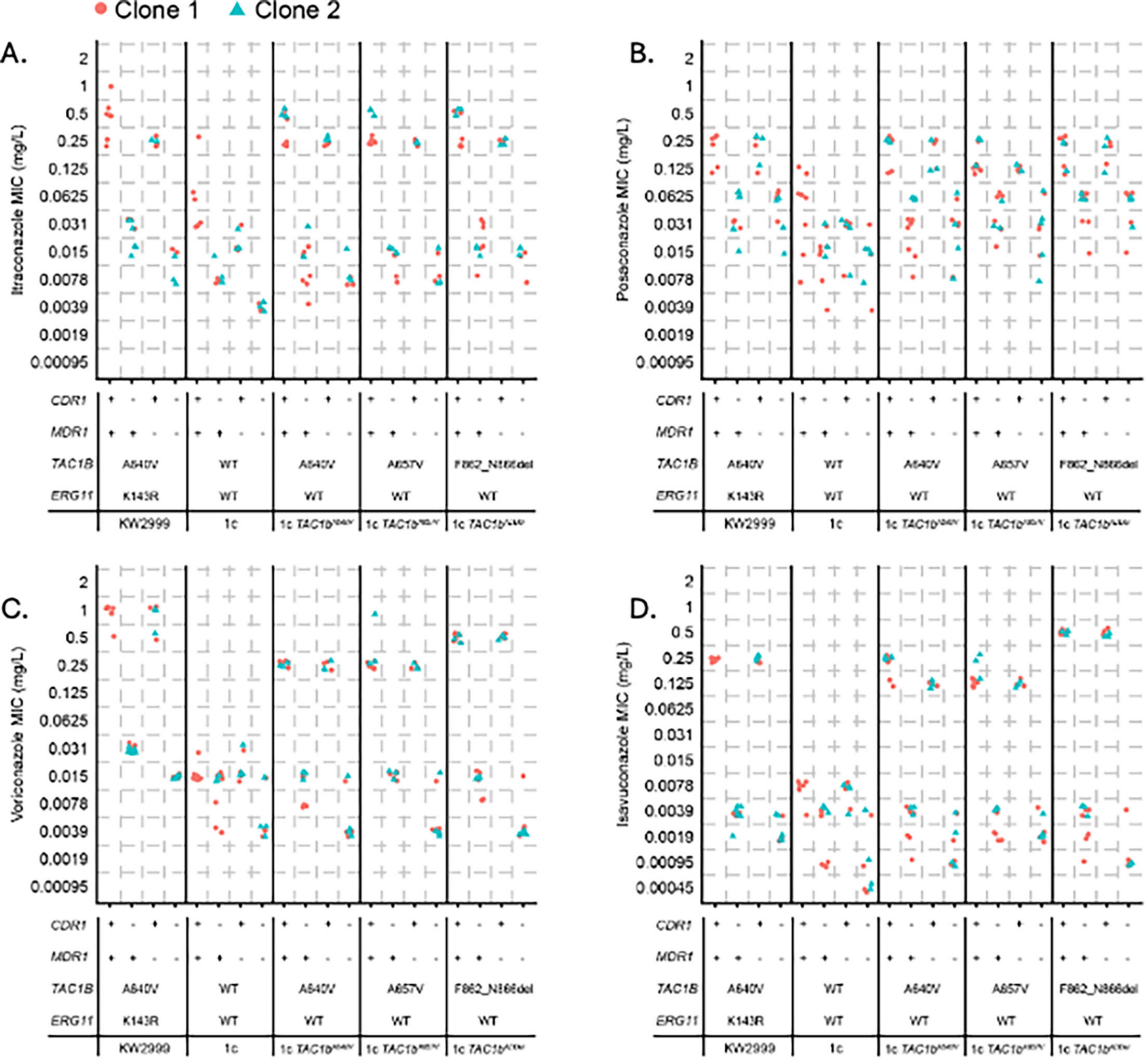
Itraconazole (**A**), posaconazole (**B**), voriconazole (**C**), and isavuconazole (**D**) MIC were measured by broth microdilution in strains in which *CDR1* and *MDR1* were disrupted by premature stop codon individually or in combination in Kw2999, 1c, or derivative backgrounds harboring indicated *TAC1B* mutations. Two independent clones were generated for each mutant combination. Each point represents a distinct MIC experimental replicate.

We also disrupted *MDR1* in the *CDR1*-disrupted derivatives of 1cA640V, 1cA657V, and 1cADdel and observed further reduction in fluconazole MICs for all strains tested. We observed a reduction from 0.5 to 0.0625–0.125 µg/mL in the 1cA640V background, 1 to 0.125 µg/mL in the 1cA657V background, and 0.5–1 to 0.0625 µg/mL in the 1cADdel background (Fig. 3). In addition, disruption of *MDR1* in *CDR1*-disrupted mutants of Kw2999 and 1c resulted in a further MIC decrease (from 8 to 1 µg/mL and from 0.25 to 0.0625 µg/mL, respectively). Similar trends for all strains were observed for itraconazole, posaconazole, voriconazole, and isavuconazole (Fig. 4). These data indicate that the majority of fluconazole resistance conferred by mutations in *TAC1B* is driven by overexpression of *CDR1*, whereas *MDR1* makes a slight contribution to fluconazole resistance in isolates carrying such mutations.

### *TAC1B, CDR1,* and *MDR1* expression are inducible by fluconazole in strains that carry the wildtype *TAC1B* allele or mutant *TAC1B***^A640V^** allele

**Fig 5:**
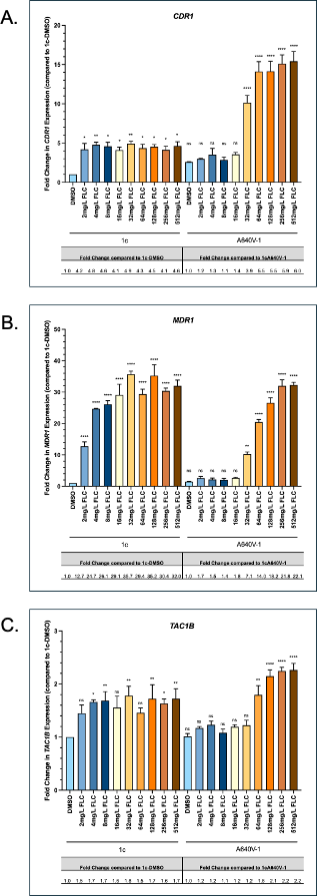
1c and 1cA640V cells were treated with indicated concentrations of fluconazole for 6 h prior to cell harvest and subsequent RNA isolation. Quantitative RT-PCR, to measure *CDR1* (**A**), *MDR1* (**B**), and *TAC1B* (**C**) RNA expression, was performed on cDNA synthesized from total RNA as described in Materials and Methods. Fold changes indicated in the bar graphs were calculated in comparison to the 1c-DMSO control sample. Fold changes reported in the table beneath each graph were calculated in comparison to each strain background’s (either 1c or 1cA640V) DMSO control. Statistical significance of pair-wise comparisons is indicated by labels above each bar: ns-not significant; *P-value 0.01–0.05; **P-value 0.01–0.001; ***P-value 0.001–0.0001; and ****P-value <0.0001.

In *C. albicans*, fluconazole exposure leads to increased expression of *CDR1* (16). Moreover, Tac1 is autoregulated as activating mutations in *TAC1* lead to its increased expression (7, 17). We therefore measured the expression of *CDR1*, *MDR1*, and *TAC1B* in strain 1c and its derivative carrying the A640V substitution in response to a range of fluconazole concentrations. Expression of all three of these genes was observed to be induced at concentrations at and above the MIC (Fig. 5). Interestingly, these genes were also induced in the strain carrying the A640V substitution. This indicates that their induction is not maximally induced by this mutation. While the fold increase in expression of *CDR1* was similar in both strains relative to their respective baseline levels of expression, presence of this mutation resulted in a maximum of 15-fold increased expression of *CDR1* relative to strain 1c.

### Disruption of *CDR1* or *MDR1* alone does not induce compensatory changes in expression of one another

**Fig 6:**
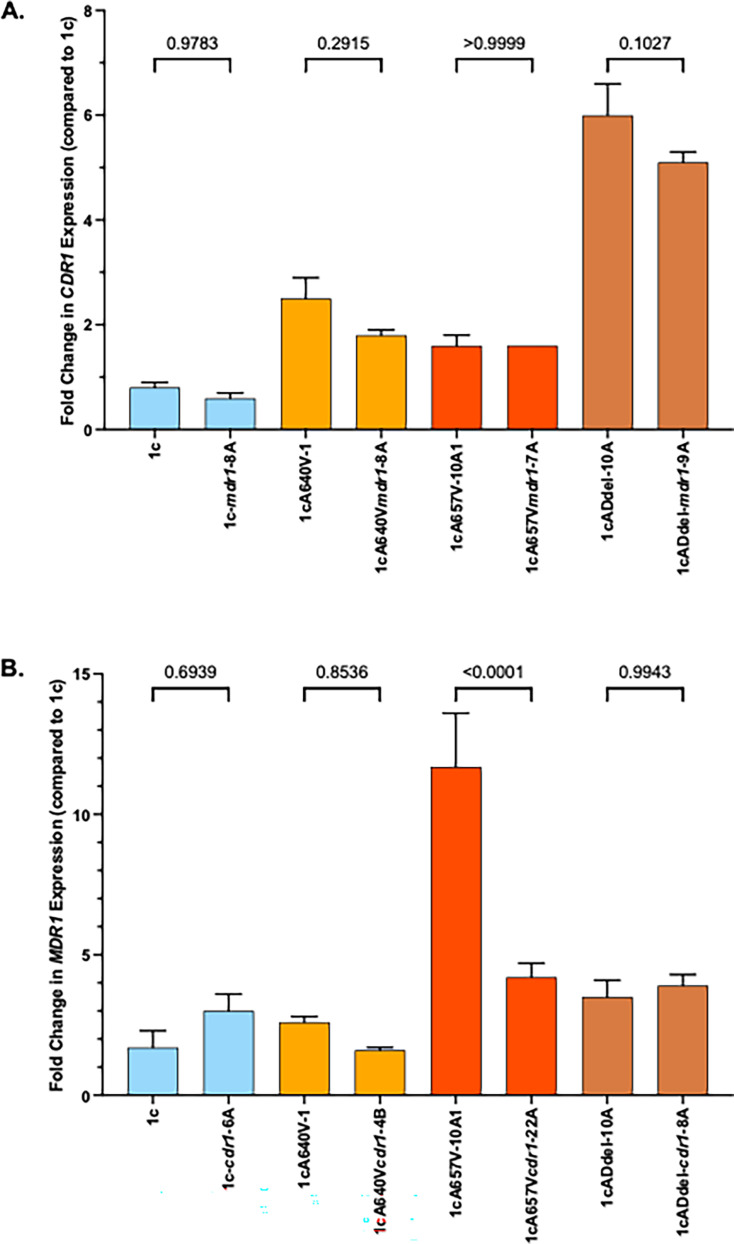
Quantitative RT-PCR was performed from cDNA synthesized from RNA isolated from 1c *TAC1B* mutant strains and their *cdr1*- and *mdr1*-disruptant derivative strains. Fold changes in *CDR1* expression (**A**) compared with strain 1c were measured in the *TAC1B* mutants and their *mdr1*-disruptant derivative strains, while fold changes in *MDR1* expression (**B**) compared with strain 1c were measured in the *TAC1B* mutants and their *cdr1*-disruptant derivative strains. Statistical significance of pair-wise comparisons is indicated by *P*-values given.

In order to determine if disruption of either *CDR1* or *MDR1* resulted in compensatory increases in expression of one another, we first measured *CDR1* expression in strain 1c and in strains carrying *TAC1B* mutations where *MDR1* had been disrupted. Disruption of *CDR1* had no effect on *MDR1* expression (Fig. 6A). Likewise, we measured *MDR1* expression in these strains where *CDR1* had been disrupted. The derivative of strain 1c that carried the mutation leading to the A657V substitution exhibited a reduction in *MDR1* expression when *CDR1* was disrupted. Otherwise, disruption of *CDR1* had no effect on *MDR1* expression (Fig. 6B).

### Overexpression of *CDR1* alone is sufficient to increase resistance to fluconazole

**Fig 7:**
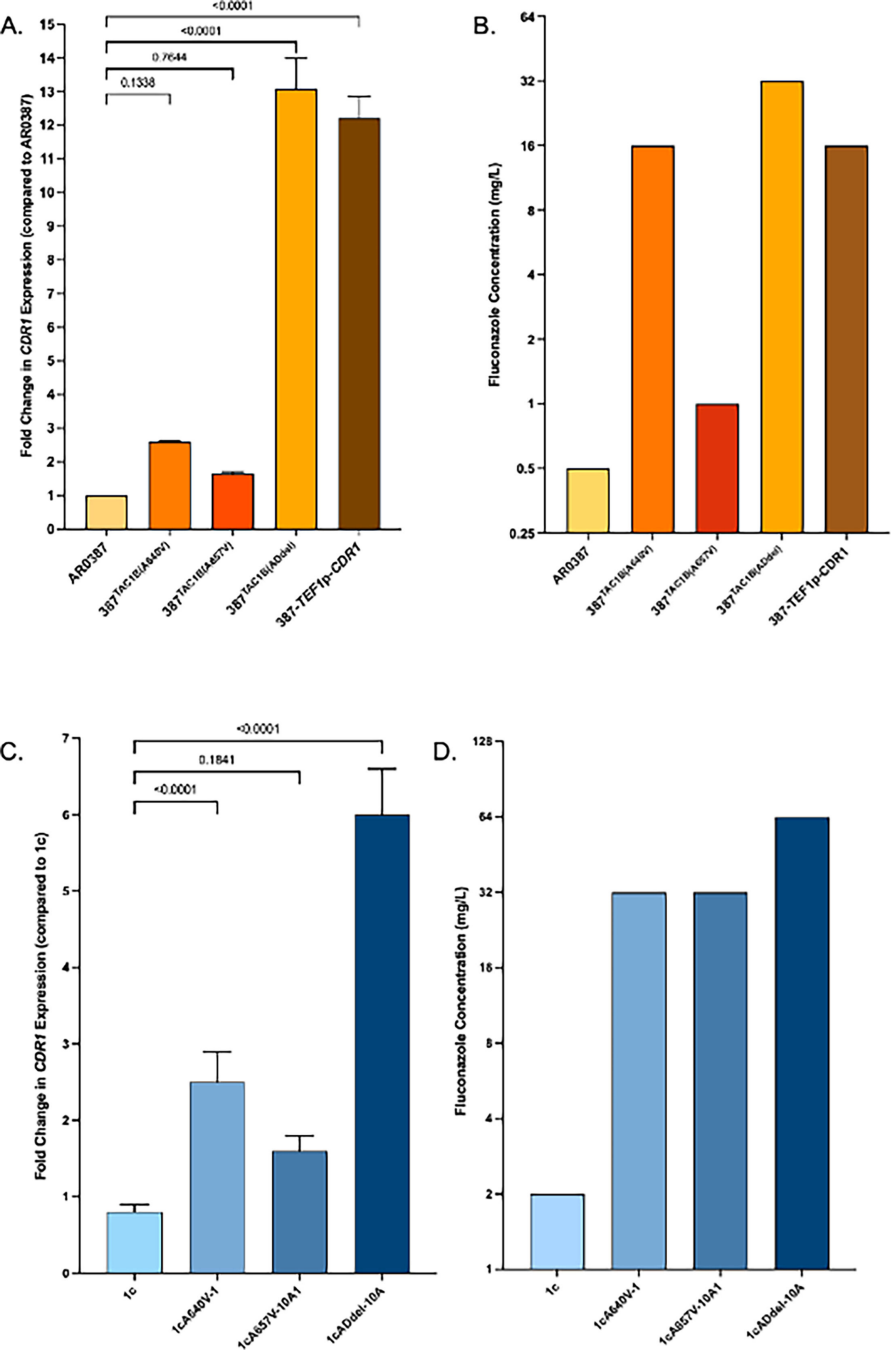
*CDR1* expression and FLC MIC were measured in *TAC1B* mutant strains or *CDR1*-overexpression strains in the AR0387 (**A and B**, respectively) or strain 1c (**C and D**, respectively) background. Fold changes in *CDR1* expression were calculated as compared to their corresponding parent controls (either isolate AR0387 or strain 1c) and pair-wise comparison *P*-values indicate statistical significance.

In order to determine if overexpression of only *CDR1* is sufficient to drive fluconazole resistance, we placed *CDR1* under the control of a strong promoter in fluconazole-susceptible isolate AR0387. We also introduced each of the three *TAC1B* mutations into this parent isolate. We observed a 12-fold increase in expression of *CDR1* which resulted in a fourfold increase in fluconazole MIC (from 1 to 16 µg/mL) (Fig. 7A and B). This level of expression and effect on MIC was similar to those observed with the mutation leading to F862_N866del. Curiously, while the level of *CDR1* expression that resulted in response to each of the *TAC1B* mutations was similar between the AR0387 and 1c backgrounds (Fig. 7C and D), the effect of the A657V substitution on fluconazole MIC was greater in the 1c background as compared with the AR0387 background.

## DISCUSSION

**Fig 8:**
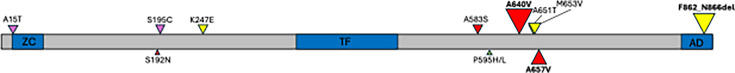
The Tac1B protein, 868 amino acids in length, is represented in gray with predicted functional domains in blue. Mutations identified in clinical isolates, as described in reference 14, are indicated along the span of the protein with the relative prevalence of each mutation indicated by the size of the triangle at the corresponding mutation position. The mutations characterized in this study are in bold. Colors of triangles indicate which clades these variants are found: red (Clade I), yellow (Clade IV), green (P595L-Clade I; P595H-Clade IV), and purple (Clade I, uniquely found together). Predicted functional domains were identified using InterPro (https://www.ebi.ac.uk/interpro/). Abbreviations: fungal Zn(2)-Cys(6) binuclear cluster domain (ZC), fungal-specific transcription factor domain (TF), and activation domain (AD).

Our findings reveal similarities and differences between genes influenced by *TAC1B* in *C. auris* and *TAC1* in the somewhat distantly related species *C. albicans*. In *C. albicans,* Tac1 regulates the genes encoding the ABC transporters Cdr1 and Cdr2, both of which contribute to fluconazole resistance through activating mutations in *TAC1* leading to upregulation of these genes (7). In *C. albicans,* Mrr1 regulates expression of the gene encoding the MFS transporter Mdr1, which also contributes to fluconazole resistance (9). In *C. auris*, *TAC1A* and *TAC1B* have been identified as homologs of *C. albicans TAC1* and are situated in tandem with 488 bp between the ORFs on chromosome 5 of the *C. auris* genome (18). Only mutations in *TAC1B* have been implicated in fluconazole resistance, and such mutations have been widely identified across resistant clinical isolates in *C. auris*, particularly in Clades I and IV (Fig. 8) (14).

We have previously shown that the A640V substitution in *TAC1B* leads to an increase in fluconazole MIC when introduced into a susceptible isolate and a reduction in MIC when the sequence is corrected to the wild-type sequence in a resistant isolate carrying this mutation (14). In the present study, we have confirmed that the mutation leading to the A640V substitution and have shown that the mutations leading to the A657V substitution and F862_N866del have similar effects on fluconazole susceptibility. While ABC transporter gene *CDR1* overexpression in response to *TAC1B* mutations observed in fluconazole-resistant isolates has not been previously established, the finding that this gene was upregulated among strains engineered to express these *TAC1B* mutations was not surprising given the relationship between *TAC1* and *CDR1* in *C. albicans* and other *Candida* species.

The overexpression of *MDR1* in strains carrying *TAC1B* mutations was unexpected and is reminiscent of the regulation of both *CDR1* and *MDR1* by Mrr1 in *C. lusitaniae* and both *CDR1B* and *MDR1B* by Mrr1 in *C. parapsilosis* (19, 20). Similar to *C. albicans*, to date, upregulation of *MDR1* in *C. auris* has only been associated with a mutation in *MRR1* present in all isolates of Clade III leading to a N647T substitution (13, 21). Introduction of this mutation into a susceptible Clade IV isolate resulted in a nearly 80-fold increase in *MDR1* expression accompanied by an increase in fluconazole MIC from 4 to 16 µg/mL and a similar fold increase in voriconazole MIC (12). Deletion of the mutant *MRR1* in a clade III isolate resulted in a reduction in MIC from 512 to 256 µg/mL and a similar fold reduction in voriconazole MIC. A similar effect was observed upon *MRR1* deletion in a different clade III isolate (18). We observed at most an 11.7-fold increase in expression of *MDR1* in response to mutations in *TAC1B*. Given that disruption of *MDR1* alone in strains carrying *TAC1B* mutations had little to no effect on fluconazole MIC, and only a modest effect when disrupted in combination with *CDR1*, this comparatively modest level of *MDR1* overexpression appears not to be sufficient to have a substantive effect on fluconazole resistance. However, the observation that disruption of both *CDR1* and *MDR1* together results in a greater increase in fluconazole susceptibility than disruption of *CDR1* alone suggests some degree of interplay between these two transporters.

We also show that expression of *CDR1*, *MDR1*, and *TAC1B* is all induced in the presence of fluconazole. This induction occurs at concentrations around the fluconazole MIC, suggesting that inhibition of lanosterol 14α-demethylase is the driver of this induction. Interestingly, we find that *CDR1* expression does not appear to be maximally induced by these mutations with proportional increases in expression in response to fluconazole leading to high-level expression in the presence of a *TAC1B* mutation. Importantly, forced overexpression of *CDR1* alone to the levels observed with the strongest of the *TAC1B* mutations is sufficient to confer a substantial increase in fluconazole MIC.

Our work establishes the role of *CDR1* regulation through *TAC1B* mutations in fluconazole resistance in *C. auris* and clarifies the role of both *CDR1* and *MDR1* in isolates carrying such mutations. A more complete understanding of the repertoire of *TAC1B* and other mutations that contribute to fluconazole resistance in *C. auris* may one day lead to genetic tools to more rapidly and accurately predict patient responses to guide selection of antifungal therapy. Moreover, understanding how these mutations lead to *CDR1* upregulation and fluconazole resistance could point to therapeutic strategies for impeding expression of this efflux pump, thereby enhancing the activity and reclaiming the utility of fluconazole against *C. auris*.

## MATERIALS AND METHODS

### Isolates, strains, and growth conditions

The clinical isolates and strains described in this study are listed in Table 2. Cells were propagated in YPD (1% yeast extract, 2% peptone, 2% dextrose) at 35°C and stored in 40% glycerol at −80°C.

**TABLE 2:** List of *C. auris* clinical isolates and derivative strains^*c*^^,^^*d*^

Isolate/Strain Name	Clone designation	Relevant genotype	Parent strain
Kw2999^*a*^	N/A	*TAC1B*^A640V^/*ERG11*^K143R^	Clinical isolate
1c	N/A	*TAC1B*^WT^/*ERG11*^WT^	Kw2999
1cA640V-1	1	*TAC1B*^A640V^/*ERG11*^WT^	1c
1cA640V-3	2	*TAC1B*^A640V^/*ERG11*^WT^	1c
1cA657V-7B1	1	*TAC1B*^A657V^/*ERG11*^WT^	1c
1cA657V-10A1	2	*TAC1B*^A657V^/*ERG11*^WT^	1c
1cADdel-10A	1	*TAC1B*^F862_N866del^/*ERG11*^WT^	1c
1cADdel-4A	2	*TAC1B*^F862_N866del^/*ERG11*^WT^	1c
2999*cdr1*-16A	1	*TAC1B*^A640V^/*ERG11*^K143R^/*CDR1*^S31*^	Kw2999
2999*cdr1*-18A	2	*TAC1B*^A640V^/*ERG11*^K143R^/*CDR1*^S31*^	Kw2999
1c-*cdr1*-6A	1	*TAC1B*^WT^/*ERG11*^WT^/*CDR1*^S31*^	1c
1c-*cdr1*-8A	2	*TAC1B*^WT^/*ERG11*^WT^/*CDR1*^S31*^	1c
1cA640V*cdr1*-4B	1	*TAC1B*^A640V^/*ERG11*^WT^/*CDR1*^S31*^	1cA640V-1
1cA640V*cdr1*-3A	2	*TAC1B*^A640V^/*ERG11*^WT^/*CDR1*^S31*^	1cA640V-1
1cA657V*cdr1*-22A	1	*TAC1B*^A657V^/*ERG11*^WT^/*CDR1*^S31*^	1cA657V-23A
1cA657V*cdr1*-23A	2	*TAC1B*^A657V^/*ERG11*^WT^/*CDR1*^S31*^	1cA657V-23A
1cADdel*cdr1*-8A	1	*TAC1B*^F862_N866del^/*ERG11*^WT^/*CDR1*^S31*^	1cADdel-6A
1cADdel*cdr1*-6A	2	*TAC1B*^F862_N866del^/*ERG11*^WT^/*CDR1*^S31*^	1cADdel-6A
2999*mdr1*-12A	1	*TAC1B*^A640V^/*ERG11*^K143R^/ *MDR1*^S5*^	Kw2999
2999*mdr1*-17A	2	*TAC1B*^A640V^/*ERG11*^K143R^/ *MDR1*^S5*^	Kw2999
1c-*mdr1*-8A	1	*TAC1B*^WT^ /*ERG11*^WT^/*MDR1*^S5*^	1c
1c-*mdr1*-17A	2	*TAC1B*^WT^ /*ERG11*^WT^/*MDR1*^S5*^	1c
1cA640V*mdr1*-8A	1	*TAC1B*^A640V^ /*ERG11*^WT^/ *MDR1*^S5*^	1cA640V-1
1cA640V*mdr1*-9A	2	*TAC1B*^A640V^ /*ERG11*^WT^/ *MDR1*^S5*^	1cA640V-1
1cA657V*mdr1*-7A	1	*TAC1B*^A657V^ /*ERG11*^WT^/*MDR1*^S5*^	1cA657V-23A
1cA657V*mdr1*-11A	2	*TAC1B*^A657V^ /*ERG11*^WT^/*MDR1*^S5*^	1cA657V-23A
1cADdel-*mdr1*-9A	1	*TAC1B*^F862_N866del^ /*ERG11*^WT^/*MDR1*^S5*^	1cADdel-6A
1cADdel-*mdr1*-12A	2	*TAC1B*^F862_N866del^ /*ERG11*^WT^/*MDR1*^S5*^	1cADdel-6A
2999*cdr1-mdr1*-7A	1	*TAC1B*^A640V^/*ERG11*^K143R^/*CDR1*^S31*^/*MDR1*^S5*^	2999*cdr1*-16A
2999*cdr1-mdr1*-10A	2	*TAC1B*^A640V^/*ERG11*^K143R^/*CDR1*^S31*^/*MDR1*^S5*^	2999*cdr1*-16A
1c-*cdr1-mdr1*-12A	1	*TAC1B*^WT^/*ERG11*^WT^/*CDR1*^S31*^/*MDR1*^S5*^	1c-*cdr1*-6A
1c-*cdr1-mdr1*-38A	2	*TAC1B*^WT^/*ERG11*^WT^/*CDR1*^S31*^/*MDR1*^S5*^	1c-*cdr1*-6A
1cA640V*cdr1-mdr1*-7A	1	*TAC1B*^A640V^/*ERG11*^WT^/*CDR1*^S31*^/*MDR1*^S5*^	1cA640V*cdr1*-3A
1cA640V*cdr1-mdr1*-11A	2	*TAC1B*^A640V^/*ERG11*^WT^/*CDR1*^S31*^/*MDR1*^S5*^	1cA640V*cdr1*-3A
1cA657V*cdr1-mdr1*-1A	1	*TAC1B*^A657V^/*ERG11*^WT^/*CDR1*^S31*^/*MDR1*^S5*^	1cA657V*cdr1*-22A
1cA657V*cdr1-mdr1*-16A	2	*TAC1B*^A657V^/*ERG11*^WT^/*CDR1*^S31*^/*MDR1*^S5*^	1cA657V*cdr1*-22A
1cADdel-*cdr1-mdr1*-3A	1	*TAC1B*^F862_N866del^ /*ERG11*^WT^/ *CDR1*^S31*^/*MDR1*^S5*^	1cADdel*cdr1*-8A
1cADdel-*cdr1-mdr1*-14B	2	*TAC1B*^F862_N866del^ /*ERG11*^WT^/ *CDR1*^S31*^/*MDR1*^S5*^	1cADdel*cdr1*-8A
AR0387	N/A	*TAC1B*^WT^/*ERG11*^WT^	Clinical isolate
AR0387_*TAC1B*^A640V*b*^	N/A	*TAC1B*^A640V^/*ERG11*^WT^	AR0387
AR0387_ *TAC1B*^A657V^	N/A	*TAC1B*^A657V^/*ERG11*^WT^	AR0387
AR0387_ *TAC1B*^ADdel^	N/A	*TAC1B*^F862_N866del^/*ERG11*^WT^	AR0387
AR0387-OE-*CDR1*	N/A	*TAC1B*^WT^/*ERG11*^WT^/*TEF*1p-*CDR1*	AR0387
AR0390	N/A	*TAC1B*^A640V^/*ERG11*^K143R^	Clinical isolate
AR0390_*TAC1B*^WT*b*^	N/A	*TAC1B*^WT^/*ERG11*^K143R^	AR0390

^*a*^ This clinical isolate was in the collection of isolates described by Ahmad et al. (15).

^*b*^ These strains were previously described by Rybak et al. (14).

^*c*^ N/A indicates not applicable.

^*d*^ * indicates that the mutation at that position results in a premature stop codon.

### Strain construction

Oligonucleotides used in this study are listed in Table 3. Construction of AR0387-OE-*CDR1* was performed as previously described (22). Briefly, the *C. auris TEF1* promoter and a nourseothricin resistance cassette were amplified from pDJS104 and assembled into the pUC19 empty vector using the GeneArt Gibson Assembly EX Master Mix (Invitrogen A46635) along with 500 bp *CDR1* homology arms targeting the *TEF1* promoter immediately upstream of *CDR1*. The assembled repair cassette was amplified as a linear fragment and electroporated into AR0387 along with a Cas9 transient expression fragment from pDJS100 and an sgRNA expression fragment targeting the *CDR1* locus generated by Splice-On-Extension PCR from pDJS101. The plasmids used in constructing this strain are described in Table 4.

**TABLE 3:** Oligonucleotides used in this study

Oligonucleotide name	Oligonucleotide sequence (5’- > 3’)^a^
Primers for AR0387-OE-CDR1 strain construction	
CDR1-OE Upstream Homology-F	acgttgtaaaacgacggccagtgaattcgagctcggtaccCAACTCTCATCTTCTCCAG
CDR1-OE Upstream Homology-R	ggggacgaggcaagcttgatGGAGATGGAAAAGTGAAG
CDR1-OE prTEF1-F	atcttcacttttccatctccATCAAGCTTGCCTCGTCC
CDR1-OE prTEF1-R	acaaaaggtttctcggacatTTTTGTTAGTTTTTGAGGTGATACAG
CDR1-OE Downstream Homology-F	cacctcaaaaactaacaaaaATGTCCGAGAAACCTTTTG
CDR1-OE Downstream Homology-R	cgccaagcttgcatgcctgcaggtcgactctagaggatccCTCATATCGTGCCAGTAATC
CDR1-OE Repair Cassette-F	CAACTCTCATCTTCTCCAG
CDR1-OE Repair Cassette-R	CTCATATCGTGCCAGTAATC
Cas9-F	CCTCTTTGTAGTTCAACTTATGC
Cas9-R	gtcccaaaaccttctcaagc
CDR1-sgRNA SOE_A-F	GCTATTACGCCAGCTGG
CDR1-sgRNA SOE_A-R	TCTCCATGTCCGAGAAACCTtggacgagtccggattc
CDR1-sgRNA SOE_B-F	CGCAATTAATGTGAGTTAGC
CDR1-sgRNA SOE_B-R	AGGTTTCTCGGACATGGAGAgttttagagctagaaatagcaag
sgDNA sequences for cloning	
TAC1B^A640WT^ TOP	ccaTCTCGTTCTTCGCCATGAAC
TAC1B^A640WT^ BOTTOM	aacGTTCATGGCGAAGAACGAGA
TAC1B^A640V^ TOP	ccaTCTCGTTCTTCGtCATGAAC
TAC1B^A640V^ BOTTOM	aacGTTCATGaCGAAGAACGAGA
TAC1B^A657V^ TOP	ccaGTTAGCAATCAAGTTCATCA
TAC1B^A657V^ BOTTOM	aacTGATGAACTTGATTGCTAAC
TAC1B^F862_N866del^ TOP	ccaCCTAATTTCTTCTTCGATAA
TAC1B^F862_N866del^ BOTTOM	aacTTATCGAAGAAGAAATTAGG
ERG11^K143(R)^ TOP	ccaCTGCTCCATCAACCTCGAGT
ERG11^K143(R)^ BOTTOM	aacACTCGAGGTTGATGGAGCAG
CDR1-STOP TOP	ccaCAACGGCAGTCTCAGTGAGG
CDR1-STOP BOTTOM	aacCCTCACTGAGACTGCCGTTG
MDR1-STOP TOP	ccaCAAAGTGTTCACATACCCCG
MDR1-STOP BOTTOM	aacCGGGGTATGTGAACACTTTG
Guide Check-F	GGGTGTCGGTTGGGTTGTG
Repair Templates (RT) and RT Amplification Primers	
ERG11^K143WT^ RT-F	GGGCACGTACCTCTGGAAAGCTTCTTTCGTCAAGGCAGTCTTAGCAAATTTCTTCTGCTCCATCAACCTCGAGTTtGGAC
ERG11^K143UNIV^ RT-R	CCGAGGCTGCTTATTCCCACTTGACCACTCCAGTTTTCGGGAAAGGTGTCATTTACGACTGTCCaAACTCGAGGTTGATG
ERG11^K143R^ RT-F	GGGCACGTACCTCTGGAAAGCTTCTTTCGTCAAGGCAGTCTTAGCAAATTTCcTCTGCTCCATCAACCTCGAGTTtGGAC
TAC1B^A640WT^ RT-F	CTTGACAGCGCAAGAACTATACTTCATCTTATTCGCGGCATCAATAGAAAAAGTGTCTCGTTCTTtGCtATGAAtTGGAT
TAC1B^A640WT^ RT-R	CGCTATCATCAGAATAATTGAGGCAGTTAGCAATCAAGTTCATCATGGCGAAAAAAGGATAAGTAATTATCCAaTTCATaGCaAAGAACGAGAC
TAC1B^A640V^ RT-F	CTTGACAGCGCAAGAACTATACTTCATCTTATTCGCGGCATCAATAGAAAAAGTGTCTCGTTCTTtGttATGAAtTGGAT
TAC1B^A640V^ RT-R	CGCTATCATCAGAATAATTGAGGCAGTTAGCAATCAAGTTCATCATGGCGAAAAAAGGATAAGTAATTATCCAaTTCATaaCaAAGAACGAGAC
TAC1B^A657V^ gBlock	TAAGAATATATCGAATGAGTTGAAGGAAGAGTTTCGTCCTCGCTTTTACTTCGAGCCTGAATTCGCGTTAATGTTGGCGAGATTTTCCGGAGA TGAAAGGATGAGCAGCGTCAATGATGGTATTCTTTCGTTCCAGCTTGCCTACTTCTTCCAGCTCATGACCATCAATAAGGTTCCTTCGCAGCT CGACCCCAATCAAGGTAATACCCCTCCTTATGAAAACTCACAGTACAGAAAACTTGCCCTTGACAGCGCAAGAACTATACTTCATCTTATTCGC GGCATCAATAGAAAAAGTGTCTCGTTCTTtGCtATGAAtTGGATAATTACTTATCCTTTTTTCGCtATGATGAACTTGATTGtTAACTGCCTCAATTAT TCTGATGATAGCGAAGGCGTTACCGACTTGAACTTGCTTATTGATTTATCAATGAACTTCTTTAATCACTACACTGATTTGGCTAAGAAACCCTC AACCGGTGCATTCTACTTACGTTTGCATTTATTTGGCATTATTATTCGTATTGCTCTTCGTATCACTGTGAAAGTATATGAAGAAAATAACAATGTT GACATATTGGGCAGCAACCCTCAGCTTAAGTCGCATTTAGAACAAGTTGAAAAGGAGTTTCCTCAATTTTACACAGAAGTGAGCGCTCCTTCC GATTTGTTGAGTCTCTTGACTTGCATGCATCCGTACACAGATATAAATCTGCAAAGTGACGACAGGAAATTCACGCCCAATGGTTC
TAC1B^A657V^ RT_Amp-F (identical to TAC1B SCN_Seq-F)	CCTCGCTTTTACTTCGAGCC
TAC1B^A657V^ RT_Amp-R	GGGCGTGAATTTCCTGTCG
TAC1B^F862_N866del^ gBlock	TCGCATTTAGAACAAGTTGAAAAGGAGTTTCCTCAATTTTACACAGAAGTGAGCGCTCCTTCCGATTTGTTGAGTCTCTTGACTTGCATGCATC CGTACACAGATATAAATCTGCAAAGTGACGACAGGAAATTCACGCCCAATGGTTCGCTTGATACATCCTCGAGCGTATCAAACCAAGACATTGC TTCGCTAACTTTTCAACACGCGAGCGGCATGGAGGCACCTTCACCCAAAAAGAATGACCCTACACTTTCCAACATACTTCATCCTGTTGACTTT GCATCTGCTAGAGATTCTGAAAGAAAAGACTTCAATATCGACGACGACCTATTGCTCGCCGCGATAAACCAAGATTTTCTGGCACTTCCTAATG GGCTTTAACTTTGTAAATAGTATGCTTACCACGATTTTAACGATGATGATATACTTTTATACTAATTCCACATATGCTAACGAGTACGAATTACGCCA GCAGCAATCAACATGTTGACTTTCTTTGCCACCTCTGGGTTCTTCATGTGCTCTTGCAATGCCGCTGGATTATCTCTGGCTTGACCCAATATACC CTGCATGACAGGGTCTTGCAAAATTTCAACTATTTCTGGGTCCTTAGACACTCTCTCCATCGTTTGTTCCGGAGTTTCGCCCTCGATTGCTGCAA AACGCTGCGACATTGCTCTGTGCATCAACTGGTTGATTTCGTTGGCGTTCTTTCCACCGTTCAACTCTTGATCCTTTGCAAGAGCG
TAC1B^F862_N866del^ RT_Amp-F	GAAGTGAGCGCTCCTTCCG
TAC1B^F862_N866del^ RT_Amp-R	CGCCAACGAAATCAACCAG
CDR1-STOP gBlock	GGCAAATTGTCCCGCTGGAAGTGGAGACTCCAGTTGTTGCCCACAGGTTATGAAGCTGGTGGTTATTTTTGTTGTATGTGGTTAGGGCTGAGAA GGGCGGTTTTTTTGTGGCATTTTGTCCCCAGTGATTAAATGTGCACCGCCAGGCTCCCGGTGGGAGTGGCCAGAGCCGGAACTCGCACCTTCC GAAGTTCCACCGCCAGCCGTCTCCAACGTGCGCAACTCTCGCACAATACGCTATTTGCCGCCAGAATTCCCTATCTCATGCCTGCTGCTCCTGCA GCGTAGCTGGGGCTGCTTCTACCTCTCCCTCTGCGACATTGTTTGGCATTTCTTTTCGCCTGCCAATATATATATCGCATGTAGTCCACTTTAAAGG CAAATCTTCACTTTTCCATCTCCATGTCCGAGAAACCTTTTGTCGACGCTCCTCCACCCGAGGATGGCGTTGCTCACCAAGTGCTGCCCCATGAC AACGGCAGTCTCAGTGctagctagctagAGGAGGCCAATTCCATCAATGAGTATACTGGTTTTGGTGCTCATCAGGAAGGTGAAATTAGAGAGTTGGCC AGAACCTTCACCAACATGTCCCATGACTCCGGCCACGACTTATCCAAAACAAACACATCCCAGGATTTGCTCAAGTACTTGTCCCACATGTCTGAG GTGCCTGGCGTAGAGCCCTTTGACCCAGAGCAGATCAGCGAGCAGTTGAACCCAGACTCGCCCAACTTCAATGCGAAGTTTTGGGTGAAAAATAT GCGTAAGTTGTTCGATTCCAACCCTGACTACTATAAGCCTTCAAAGTTGGGACTTGCGTACCGTAATTTGAGAGCCTACGGTGTGGCTGCAGACTC AGACTACCAGCCAACCGTCAGTAACGGGTTGTGGAAAATGGCGGTGGATTACTGGCAC TCTTAAAGACCATGGACGGGTACTTCAAGCCCGGT
CDR1 RT_Amp-F	CCGCTGGAAGTGGAGACTCC
CDR1 RT_Amp-R	GCTTGAAGTACCCGTCCATGG
MDR1-STOP gBlock	GGTTCACGTAAGCGACCCTTCGTACGGTCCTGTCAATATGCAGCACGTACCCGTCGTTCGGCGCCTACCATTTTAACCCGGAGATGCGACAAGGG CACAAAAAGCGACTAGTCATTGCGCAAGCAAACGTGTGAAAGTGACTCTAAAAGACTGATTTAGGGAGACCTGGAAGGAGTTCGAAAAGCTCATG AAACTAAAAAATAAAATGAATAATTAAAAAAAAAAAAAAAAAGCAATGCCTCGTACTCCCGTCAAACTCACGTAAGCCTTTTCGTCTGCACCCAAACA AAGACAATCTAACACTTTCGTACATTTTCTGCGCGGCTTAACTCAAATGTGGCGGCTGCAAAAGGTATGCCATTTTTGCTCTCATTACTCATCAAAAA AATAGTGATAAGGTACACTGAGACATATAAATAGGCTGTAACTTCTCTGATTCTTTCGTCTTTACCAAATTTCAACGCATCTTCAATCTCCACATGTTC CTagcTAgcTagGTCAGAGAGAGCTTCTTCGGCAGGTCTCTATATCACTTATCTGGACGCAAAGTGTTCACATACCCCGAGGAATCACCCGACTATGT GATTCCCGCAAAGTACTTGGGCAAAGACGAAGCAGGCATTGAATCGGATGTTCAGGAGAAAGCTGGCGCTTCAGACACCCCCGTTGATCTGGAC TCTTCGTCCCAGTCGACCAAGACCAACCACATTCTTGTGGACTGGGAGGGAGAAGACGATCCAGAAAATCCATACAATTGGCCATTGAAATACAAG ATTATCTTCATTGCCCAGATCATGATTTTGACTGCATTTGTGTATATGGCTTCTGCCATTT GTGGGCCAGGTGGTGGCGACACTCCCCTTGACGCTTTTTGTGTTCGGATACGGTATCGGGCCCATGGTGTTTTCGCCGCTTTCGGAAAATGCCA GGTTTGGCAGAACGTCCATCTACATCATTACCTTATTTATCTTCTTTATCTTGCAGATCCCCACGGCTCTCGT
MDR1 RT_Amp-F	GCGACCCTTCGTACGGTC
MDR1 RT_Amp-R	CCGTGGGGATCTGCAAG
Screening and sequencing primers	
TAC1B SCN_Amp-F	CAAGTTGTCATCTCGGCCATC
TAC1B SCN_Amp-R	CAACATGTTGATTGCTGCTGGC
TAC1B SCN_Seq-F	CCTCGCTTTTACTTCGAGCC
TAC1B SeqF1	CCTCCTGCGCCTTCCAC
TAC1B SeqF2	CAACAACGCTTCCAATGAGCC
TAC1B SeqF3	GAGTTACCGATCTGCGCCCC
TAC1B SeqF4	CACCTCTCTTAACGGCGAAC
TAC1B SeqF5	CATATTGGGCAGCAACCCTC
CDR1 5’ SCN_Amp-F	GAGGCAGCCCACTGTATGG
CDR1 ORF SCN_Amp-R	GTGGCATCCAAGATGGCCG
CDR1-STOP SCN_Seq	GCTTGAAGTACCCGTCCATGG
ERG11 SCN_Amp-F	CTCTTGGACCAAAAACAGCCAGC
ERG11 SCN_Amp-R	GGCTGGAGCTGGTTTGGTG
ERG11 SCN_Seq-F	GCAGAGAGAAATACGGCGATGTG
ERG11 SeqF1	CCATCGTATAGTGGTCCTCC
ERG11 SeqF2	GGGACTTGATCGACTCGC
MDR1-STOP SCN_Amp-F	CGGCCGTATATGTGGGCTC
MDR1-STOP SCN_Amp-R	CCCGTGAGAGCTCTGAGTC
MDR1dis SCN_SeqF	GGGCGATTCTCCGGTGG
qRT-PCR primers	
ACT1 qRTPCR-F	GAAGGAGATCACTGCTTTAGCC
ACT1 qRTPCR-R	GAGCCACCAATCCACACAG
CDR1 qRTPCR-F	GAAATCTTGCACTTCCAGCCC
CDR1 qRTPCR-R	CATCAAGCAAGTAGCCACCG
MDR1 qRTPCR-F	GAAGTATGATGGCGGGTG
MDR1 qRTPCR-R	CCCAAGAGAGACGAGCCC

^a^ Lower-case nucleotides represent nucleotide changes from the original sequence.

**TABLE 4:** Plasmids used in AR0387-OE-*CDR1* strain construction

Plasmid (Alias)	Description	Source
pDJS100 (pTO135) – pCauCas9	Vector harboring *C. auris* transient Cas9 expression cassette	(22)
pDJS101 (pTO136) – pCausgRNA	Vector harboring *C. auris* transient sgRNA expression cassette	(22)
pDJS104 (pTO288)	Vector harboring repair cassette containing NAT-prTEF1	(23)

*C. auris* strain construction for all other strains was based on the methods described previously (24, 25) and described here briefly:

- *Repair template design and preparation*. Forward and reverse primers for *TAC1B*^A640^ (wild type), *TAC1B*^A640V^, *ERG11*^K143^ (wild type), and *ERG11*^K143R^ manipulation, corresponding to the sequence surrounding the manipulation site with a silent mutation to destroy the PAM site and either containing the sequence resulting in the wildtype or mutant amino acid at the same position, were designed with a 20 bp overlap. Repair templates were prepared with these primers by primer extension (one cycle of 94°C, 5 min, 35 cycles of 94°C, 30 s/42°C, 45 s/72°C, 30 s, and one cycle of 72°C for 5 min) in three 100 µL reactions containing 3 nmol each primer in 1× Phusion Green master mix (Invitrogen). The repair templates used to introduce the *TAC1B*^A657V^ or *TAC1B*^F862_N866del^ mutations to the wild-type sequence corresponded to 362 bp upstream and 387 bp downstream of the manipulation site for the A657V substitution, and 381 bp upstream and 366 bp downstream for the F862_N866del in-frame deletion, were synthesized as gBlocks by Integrated DNA Technologies (Coralville, IA). The repair template used for disruption of the *MDR1* ORF was synthesized as a gBlock corresponding to sequence 518 bp upstream of the manipulation site, a stop codon cassette consisting of 5′-TAGCTAGCTAG-3′ replacing the original sequence 7 bp downstream of the start codon, and sequence 521 bp downstream of the manipulation site. Similarly, the repair template used for disruption of the *CDR1* ORF was synthesized as a gBlock corresponding to sequence 490 bp upstream of the manipulation site, the stop codon cassette replacing the original sequence 88 bp downstream of the start codon, and sequence 488 bp downstream of the manipulation site. The gBlock repair templates were amplified using standard PCR conditions using Phusion Green master mix (Invitrogen/Thermo Fisher Scientific; Waltham, MA). PCR products were purified using a Qiagen PCR Purification kit per manufacturer’s instructions and eluted in 25 µL nuclease-free water (QIAGEN; Hilden, Germany).
- *C. auris transformations*. Forty milliliters of an overnight culture initiated by inoculating YPD with a single colony of *C. auris* (final OD_600_ = 1.2–1.8) was used to prepare electrocompetent cells as described previously (13). One microgram of guide-specific plasmid and 5 µg repair template were combined with 40 µL electrocompetent cells in a 0.2-cm BioRad Gene Pulser cuvette, and *C. albicans* programmed Gene Pulser settings were used to electroporate the cells (Bio-Rad Laboratories; Hercules, CA). Cells were allowed to recover in 0.5 mL 1M sorbitol + 1 mL YPD for 5 hrs at 35°C then spread on YPD + 200 µg/mL nourseothricin agar plates.
- *Screening for positive transformants*. Initial screening of colonies from transformation plates was performed by colony PCR in 50 µL reactions containing 1× Phire Plant master mix (Invitrogen/Thermo Fisher Scientific) and using primers that bind sequences upstream and downstream of the repair template sequence. Purified PCR products were subjected to Sanger sequencing with locus-specific primers to detect the desired nucleotide change(s). Positive transformants were cultured in 5 mL YPD at 35°C for up to three passages of up to 72 h each to allow the cells to be cured of the pJMR19 plasmid. An aliquot of the culture was diluted 10^4^ and plated on YPD agar plates. Resulting colonies were replica plated on YPD agar and YPD + 200 µg/mL nourseothricin agar plates to screen for nourseothricin-sensitive colonies. Final confirmation of the entire sequence of the target gene was confirmed by amplifying the entire ORF plus ~200 bp of flanking sequence with locus-specific primers from genomic DNA isolated using MasterPure Yeast DNA Purification kit (LGC Biosearch Technologies; Middlesex, UK) in 50 µL reactions containing 1× Phusion Green master mix and subjecting the purified PCR products to Sanger sequencing.

### Minimum inhibitory concentration (MIC) determinations by broth microdilution

MICs for fluconazole, itraconazole, voriconazole, posaconazole, and isavuconazole were measured by modified CLSI broth microdilution assays as described previously (11).

### RNA isolation

An aliquot of cells from an overnight culture was used to inoculate 10 mL MOPS-buffered RPMI + 2% glucose (pH 7.0) to an OD_600_ = 0.08–0.12, followed by incubation at 35°C in a shaking incubator (220 rpm) until mid-log phase (6 h). Cell cultures were grown in triplicate. Cells were collected by centrifugation, supernatants removed, and cell pellets stored at −80°C. RNA was extracted using methods described previously with some modification (22). Cell pellets were resuspended in 100 µL FE Buffer (98% formamide, 0.01 M EDTA) at room temperature. Fifty microliters of 1 mm RNase-free glass beads was added to the cells and were subjected to vortex disruption for 5 min at room temperature followed by snap-cooling on ice. The cell lysate was clarified by centrifugation, and the supernatant was DNase-treated for 30 min followed by isopropanol precipitation. RNA integrity was confirmed by agarose gel electrophoresis, and concentrations were approximated by Nanodrop.

### cDNA synthesis and qRT-PCR

cDNA was synthesized from 500 ng RNA using the RevertAid First Strand cDNA Synthesis Kit (Invitrogen/Thermo Fisher) with the provided random primer mix according to the manufacturer’s instructions. qRT-PCR was performed from three biological replicates, each with three technical replicates, using SYBR Green PCR master mix (Bio-Rad). Fold changes were calculated using the △△^CT^ method with target gene CT values normalized to *ACT1* CT values (generating dCT values) and the median 1c dCT value (from the three biological replicates) for each target gene used as the comparator for fold change calculation.

### RNA sequencing and analysis

RNA sequencing was performed using Illumina NextSeq for stranded mRNA. Libraries were prepared with paired-end adapters using Illumina chemistries per manufacturer’s instructions, with read lengths of approximately 150 bp with at least 50 million raw reads per sample. Data were analyzed using the Galaxy web platform public server at *usegalaxy.org (*26). Read quality was assessed, and reads were quality-trimmed with a PHRED cutoff of 20 using FastP (27). Reads were then mapped to the *C. auris* B8441 reference assembly (NCBI GCA_002759435.2) using RNA Star with default parameters (28) followed by quantification using feature Counts (29) and assessment of differential expression using DESeq2 (30). Expression fold-change and significance cutoffs of >2-fold up- or downregulated and an adjusted *P*-value less than 0.05 were established to categorize dysregulated genes. Gene Ontology annotations were retrieved from the *Candida* Genome Database (31) to identify significantly dysregulated GO terms using GoSeq (32), normalizing for feature lengths extracted from the featureCounts output. Transcriptional overlap between strains was assessed using either UpsetR R Package (33) or DiVenn 2.0 (34).

## Data Availability

The GEO accession number corresponding to the RNA-seq data were assigned as GSE288372.
