# Correction for Barker et al., “Mutations in *TAC1B* drive *CDR1* and *MDR1* expression and azole resistance in *Candida auris*”

**DOI:** 10.1128/aac.01507-25

**Published:** 2026-06-04

**Authors:** Katherine S. Barker, Darian J. Santana, Qing Zhang, Tracy L. Peters, Jeffrey M. Rybak, Joachim Morschhäuser, Christina A. Cuomo, P. David Rogers

## AUTHOR CORRECTION

Volume 69, no. 10, e00300-25, 2025, https://doi.org/10.1128/aac.00300-25. Throughout the article, “fold change in gene expression” should read “relative gene expression.”

Figure 2: The *y* axis of panel A should read “Relative *CDR1* Expression Level (compared to median 1c dCT),” the *y* axis of panel B should read “Relative *MDR1* Expression Level (compared to median 1c dCT),” the *y* axis of panel C should read “Relative *CDR1* Expression Level (compared to median AR0387 dCT),” and the *y* axis of panel D should read “Relative *MDR1* Expression Level (compared to median AR0387 dCT).”

Figure 5: The *y* axis of panel A should read “Relative *CDR1* Expression Level (compared to median 1c-DMSO dCT),” the *y* axis of panel B should read “Relative *MDR1* Expression Level (compared to median 1c-DMSO dCT),” and the *y* axis of panel C should read “Relative *TAC1B* Expression Level (compared to median 1c-DMSO dCT).”

Figure 6: The *y* axis of panel A should read “Relative *CDR1* Expression Level (compared to median 1c dCT)” and the *y* axis of panel B should read “Relative *MDR1* Expression Level (compared to median 1c dCT).”

Figure 7: The *y* axis of panel A should read “Relative *CDR1* Expression Level (compared to median AR0387 dCT)” and the *y* axis of panel C should read “Relative *CDR1* Expression Level (compared to median 1c dCT).”

Results, “Disruption of CDR1 or MDR1 alone does not induce compensatory changes in expression of one another”: “Disruption of *CDR1* had no effect on *MDR1*...” should read “Disruption of *MDR1* had no effect on *CDR1*...”

Figure 8 caption: “…indicate which clades these…” should read “… indicate the clades in which these…”

Materials and Methods, “cDNA synthesis and qRT-PCR”: “…values) and the 1c median dCT value (from the three biological replicates) for each target gene used as the comparator for fold change calculation” should read “…values) and the median dCT value of the comparator strain (from the three biological replicates) subtracted from each sample’s dCT to generate ddCT values. Relative gene expression levels were expressed as 2^-ddCT^.”

